# Publisher Correction: Second-order spectral lineshapes from charged interfaces

**DOI:** 10.1038/s41467-017-02179-8

**Published:** 2018-01-15

**Authors:** Paul E. Ohno, Hong-fei Wang, Franz M. Geiger

**Affiliations:** 1 Department of Chemistry, Northwestern University, Evanston, IL 60208 USA; 2 Department of Chemistry and Shanghai Key Laboratory of Molecular Catalysis and Innovative Materials, Fudan University, Shanghai, 200433 China

*Nature Communications* 10.1038/s41467-017-01088-0, Article published online 18 October 2017

The original version of this Article contained an error in Equation 3b. A ‘ + ’ sign incorrectly appeared instead of a ‘–’ sign in the denominator of the right-hand side of the equation and incorrectly read:

$$\left( {\chi _1^{\left( 3 \right)} + i\chi _2^{\left( 3 \right)}} \right) = \left( {\frac{{\kappa ^2}}{{\kappa ^2 + \left( {\Delta k_z} \right)^2}}\chi ^{\left( 3 \right)} + i\frac{{\kappa \Delta k_z}}{{\kappa ^2 + \left( {\Delta k_z} \right)^2}}\chi ^{\left( 3 \right)}} \right) = \frac{\kappa }{{\kappa + i\Delta k_z}}\chi ^{\left( 3 \right)}$$

The correct form of the equation is as follows:

$$\left( {\chi _1^{\left( 3 \right)} + i\chi _2^{\left( 3 \right)}} \right) = \left( {\frac{{\kappa ^2}}{{\kappa ^2 + \left( {\Delta k_z} \right)^2}}\chi ^{\left( 3 \right)} + i\frac{{\kappa \Delta k_z}}{{\kappa ^2 + \left( {\Delta k_z} \right)^2}}\chi ^{\left( 3 \right)}} \right) = \frac{\kappa }{{\kappa - i\Delta k_z}}\chi ^{\left( 3 \right)}$$

This has now been corrected in both the PDF and HTML versions of the Article.

