# Supplementary material for: Second-order spectral lineshapes from charged interfaces
Source: Nat Commun. 2017 Oct 18;8:1032. doi: 10.1038/s41467-017-01088-0 (PMC5647331; doi:10.1038/s41467-017-01088-0)
Supplement: Supplementary file 1 — Supplementary Information [file 41467_2017_1088_MOESM1_ESM.pdf]

Supplementary Note 1

A brief derivation of the origin of the $i\chi_2^{(3)}$ term for conditions of absorptive-dispersive mixing.

To be consistent with the literature[2,3], we modify here the phase matching factor from the one we used in our previous derivation[1] ($e^{-i\Delta k_z z}$) to $e^{i\Delta k_z z}$. Importantly, we found in the SFG spectral simulation that only the $e^{i\Delta k_z z}$ form gives a correct calculation of the SFG interference lineshape from the values of Δk_z and κ.

As the electric field $E_{dc}(\mathrm{z}) = -d\Phi(\mathrm{z})/\mathrm{dz}$ is z (depth)-dependent and there is the phase matching factor that is also z dependent, one has:

$$\begin{aligned}
\chi_{dc}^{(2)} &= \int_0^\infty \chi^{(3)} E_{dc}(z) e^{i\Delta k_z z} dz \\
&= \int_0^\infty -\chi^{(3)} \frac{d\Phi(\mathrm{z})}{dz} e^{i\Delta k_z z} dz \\
&= -\chi^{(3)}\Phi(\mathrm{z})\,\mathrm{e}^{i\Delta k_z z}\Big|_0^\infty + \chi^{(3)}\int_0^\infty \Phi(\mathrm{z})(i\Delta k_z) e^{i\Delta k_z z} dz \\
&= \chi^{(3)}\Phi(0) + i\Delta k_z \chi^{(3)} \int_0^\infty \Phi(\mathrm{z}) e^{i\Delta k_z z} dz
\end{aligned} \quad (1)$$

Here, $1/\Delta k_z$ is the coherence length of the SHG or SFG process, $\Phi(\infty) = 0$, and the following integration relationship was used:

$$\int \frac{df(\mathrm{z})}{dz} g(\mathrm{z})\,\mathrm{dz} = f(z)g(z) - \int f(z)\frac{dg(\mathrm{z})}{dz} dz \quad (2)$$

A good approximation is that $\Phi(z) = \Phi(0)\,\mathrm{e}^{-\kappa z}$, where 1/κ is the Debye screening length factor. Then,

$$\begin{aligned}
\chi_{dc}^{(2)} &= \chi^{(3)}\Phi(0) + i\Delta k_z \chi^{(3)} \int_0^\infty \Phi(0) e^{-\kappa z} e^{i\Delta k_z z} dz \\
&= \chi^{(3)}\Phi(0) + \frac{i\Delta k_z}{\kappa - i\Delta k_z}\chi^{(3)}\Phi(0) \\
&= \frac{\kappa}{\kappa - i\Delta k_z}\chi^{(3)}\Phi(0)
\end{aligned}$$

Therefore, in the total effective surface susceptibility,

$$\chi_{eff}^{(2)} = \chi^{(2)} + \chi_{dc}^{(2)} = \chi^{(2)} + (\chi_1^{(3)} + i\chi_2^{(3)})\Phi(0) \quad (3)$$

one has

$$\chi_1^{(3)} = \frac{\kappa^2}{\kappa^2 + (\Delta k_z)^2}\chi^{(3)} \quad (4)$$

$$\chi_2^{(3)} = \frac{\kappa\Delta k_z}{\kappa^2 + (\Delta k_z)^2}\chi^{(3)} \quad (5)$$

Therefore, because the surface field is real and the phase matching factor is complex, the total $\chi_{dc}^{(2)} = (\chi_1^{(3)} + i\chi_2^{(3)})\Phi(0)$ contribution is complex.

When $\kappa << \Delta k_z$, i.e. the Debye length is long (low electrolyte concentration), one finds

$$\chi_1^{(3)} \sim 0 \text{ and } \chi_2^{(3)} \sim \frac{\kappa}{\Delta k_z}\chi^{(3)} \qquad (6)$$

and the *dc* contribution is essentially imaginary.

When $\kappa >> \Delta k_z$, i.e. the Debye length is very small (high electrolyte concentration), one finds

$$\chi_1^{(3)} \sim \chi^{(3)} \text{ and } \chi_2^{(3)} \sim \frac{\Delta k_z}{\kappa}\chi^{(3)} \sim 0 \qquad (7)$$

and the real term dominates.

When $\kappa \sim \Delta k_z$, i.e. the Debye length and phase matching coherence length are comparable, the real and imaginary terms for the $\chi^{(3)}$ are comparable.

The derivation above assumes that the surface potential is of the form $\Phi(z) = \Phi(0)e^{-\kappa z}$. The actual surface potential may be different from this form, but essentially it decays when moving away from the surface. In addition, the surface potential can not only induce bulk $\chi^{(3)}$ responses from the water side, but also from the fused silica or the α-quartz side.[4] These issues warrant further investigation in the future. Nevertheless, the following relationship, as established herein, should generally hold:

$$\chi_{eff}^{(2)} = \chi^{(2)} + \chi_{dc}^{(2)} = \chi^{(2)} + (\chi_1^{(3)} + i\chi_2^{(3)})\Phi(0) \qquad (8)$$

Supplementary References

1. Ohno, P. E., Saslow, S. A., Wang, H.-f., Geiger, F. M. & Eisenthal, K. B. Phase-referenced Nonlinear Spectroscopy of the alpha-Quartz/Water Interface. *Nature communications* **7**, 13587 (2016).
2. Wen, Y.-C., Zha, S., Liu, X., Yang, S., Guo, P., Shi, G., ... & Tian, C. Unveiling Microscopic Structures of Charged Water Interfaces by Surface-Specific Vibrational Spectroscopy. *Phys. Rev. Let.* **116**, 016101 (2016).
3. Gonella, G., Lutgebaucks, C., de Beer, A. G. F. & Roke, S. Second Harmonic and Sum-Frequency Generation from Aqueous Interfaces is Modulated by Interference. *J. Phys. Chem. C* **120**, 9165-9173 (2016).
4. Bethea, C. G. Electric field induced second harmonic generation in glass. *Appl. Optics* **14**, 2435-2437 (1975).
