# Supplementary material for: Second-order spectral lineshapes from charged interfaces
Source: Nat Commun. 2017 Oct 18;8:1032. doi: 10.1038/s41467-017-01088-0 (PMC5647331; doi:10.1038/s41467-017-01088-0)
Supplement: Supplementary file 3 — Description of Additional Supplementary Files [file 41467_2017_1088_MOESM3_ESM.pdf]

Description of Additional Supplementary Files

File Name: Supplementary Data 1
Description: Mathematica notebook for generating the data shown in Figure 1a-c.

File Name: Supplementary Data 2
Description: Mathematica notebook for generating the data shown in Figure 2a-b.

File Name: Supplementary Data 3
Description: Mathematica notebook for generating the data shown in Figure 3.

File Name: Supplementary Data 4
Description: Mathematica notebook for generating the data shown in Figure 4a-d

File Name: Supplementary Data 5
Description: Mathematica notebook for generating the data shown in Figure 4e-f.

File Name: Supplementary Data 6
Description: Mathematica notebook for generating the data shown in Figure 5-6.

File Name: Supplementary Data 7
Description: Mathematica function the interpolation of the refractive index of water.
